# Dynamics of carbon and CO_2_ removals by Brazilian forest plantations during 1990–2016

**DOI:** 10.1186/s13021-018-0106-4

**Published:** 2018-10-22

**Authors:** Carlos Roberto Sanquetta, Ana Paula Dalla Corte, Allan Libanio Pelissari, Margarida Tomé, Greyce Charllyne Benedet Maas, Mateus Niroh Inoue Sanquetta

**Affiliations:** 1 BIOFIX Research Center, Federal University of Paraná, Curitiba, Paraná Brazil; 2 University of Lisbon, FORCHANGE Research Group, Lisbon, Portugal

**Keywords:** Biomass, Greenhouse gases, Mitigation, Planted forests, Wood products

## Abstract

**Background:**

We analyzed the dynamics of carbon (C) stocks and CO_2_ removals by Brazilian forest plantations over the period 1990–2016. Data on the extent of forests compiled from various sources were used in the calculations. Productivities were simulated using species-specific growth and yield simulators for the main trees species planted in the country. Biomass expansion factors, root-to-shoot ratios, wood densities, and carbon fractions compiled from literature were applied. C stocks in necromass (deadwood and litter) and harvested wood products (HWP) were also included in the calculations.

**Results:**

Plantation forests stocked 231 Mt C in 1990 increasing to 612 Mt C in 2016 due to an increase in plantation area and higher productivity of the stands during the 26-year period. Eucalyptus contributed 58% of the C stock in 1990 and 71% in 2016 due to a remarkable increase in plantation area and productivity. Pinus reduced its proportion of the carbon storage due to its low growth in area, while the other species shared less than 6% of the C stocks during the period of study. Aboveground biomass, belowground biomass and necromass shared 71, 12, and 5% of the total C stocked in plantations in 2016, respectively. HWP stocked 76 Mt C in the period, which represents 12% of the total C stocked. Carbon dioxide removals by Brazilian forest plantations during the 26-year period totaled 1669 Gt CO_2-_e.

**Conclusions:**

The carbon dioxide removed by Brazilian forest plantations over the 26 years represent almost the totality of the country´s emissions from the waste sector within the same period, or from the agriculture, forestry and other land use sector in 2016. We concluded that forest plantations play an important role in mitigating GHG (greenhouse gases) emissions in Brazil. This study is helpful to improve national reporting on plantation forests and their GHG sequestration potential, and to achieve Brazil’s Nationally Determined Contribution and the Paris Agreement.

## Background

It is strategically important for any country to have up-to-date and reliable information on the state and future trends of its forests since good forestry statistics are essential tools to implement public policies to attract productive investments. Moreover, forestry statistics are needed to guide the necessary actions to meet the international commitments assumed by the country, particularly on the intensely-debated topic of climate change.

Every 5 years, Brazil provides its forestry information to the FAO (Food and Agriculture Organization of the United Nations), reporting wood volume, biomass, and carbon stocks, among other data [1]. The sectoral statistics provided by the SFB (Brazilian Forest Service) to the FAO are based on secondary and indirect data, due to the lack of primary information. In future, this data gap may be filled by the completion of the new forest inventory that has been carried out in various regions of the country [2].

Brazil’s forest statistics reported in FRA (Forest Resources Assessment) reports [1] have shown a methodological evolution, due to the many studies carried out in recent years by Brazilian [3–8] and overseas researchers and institutions. Improving the quality of information is essential to ensure FRAs can be accurately used for accounting purposes. Among the most relevant data to policymakers are biomass and carbon stocks, as well as their temporal dynamics. These data can be used by the forestry industry as well as by the government committed to its Nationally Determined Contribution (NDC), a requirement of the Paris Agreement [9].

Despite having the second largest area of natural forests, and the eighth largest area of forest plantations in the world [1, 10], Brazil still has a modest share of the global forest economy. One of the reasons may be the lack of robustness and quality of Brazil’s forest information in the long term, compatible with the profile of forestry. Efforts have been made by SFB to improve forestry data through a continuous process of updating the National Information System [2].

Currently, forest plantations account for 85% of the wood consumed in Brazil [11], which corroborates the importance of having updated and reliable information about their extent and growth stocks. Plantations are essentially composed of fast-growing exotic species that have adapted well to local environmental conditions and proven to have excellent technological applications. The main planted species belong to the Eucalyptus and Pinus genera, which have been considered among the most productive in the world [12], with an MAI (mean annual increment) over 40 and 30 m^3^ ha^−1^ year^−1^, respectively. More recently, alternative species have also appeared in the portfolio, including Teak (*Tectona grandis*) and Acacias (*Acacia mearnsii*, *A. mangium*). The wood coming from such plantations serves to meet the strong demand for pulp and paper, sawnwood, wood-based panels, steel, and finished products.

Forest plantations, besides supplying raw materials for forest-based mills, also contribute to the mitigation of climate change by storing atmospheric carbon in their biomass. However, Brazil is notable in this context, since it is considered a major emitter of greenhouse gases (GHG) from the agriculture, forestry, and other land uses (AFOLU) sector. Despite this, AFOLU activities can play an important role in climate mitigation [13], especially in relation to forestry activities in Brazil.

Plantations not only help to mitigate climate change by storing carbon in their biomass and in wood products, but they also avoid emissions from other potentially more GHG-emitting materials. Thus, forest products are an alternative to reduce the effects of climate change and environmental degradation in general [14]. Additional actions involve measures to prevent deforestation, promote sustainable forest management, and encourage the use of this lumber in buildings and furniture, as well as replacing fossil fuels with wood biomass.

Although the role of Brazil’s forest plantations in climate change mitigation has been well evidenced, biomass and carbon estimates still require substantial refinement. It is necessary to reduce the degree of uncertainty in forestry data to support the accomplishment of the goals imposed by international climate agreements to which Brazil is a signatory. Methodological tiers used in the Third National Communication [15] and other GHG accounting systems [16] are quite mature for natural forests, but the specific conditions of planted forests are neglected. This work aims to fill this data gap, by quantifying the biomass and carbon stocks in Brazilian forest plantations over a broad temporal range (26 years) and demonstrating their role in mitigation of Brazil’s GHG emissions.

## Methods

### Extent of forest plantations

Three data sources on the extent of the forest plantations in Brazil were used:

1. Online data on forest yield and silviculture, named PEVS managed by IBGE [11];
2. The 2017 report of the Brazilian Forestry Industry Association (IBÁ) [12];
3. The Global Forest Resources Assessment—FRA2015 Brazil [1].

The data from PEVS refers to the period 2014–2016 and are considered official statistics since they were produced and published by the office responsible for Brazil’s main statistics (IBGE). The data in the second reference corresponds to the period 2010–2016 and were produced by the institution that represents forestry companies. FRA2015 is the report submitted by the SFB to FAO, which publishes it every 5 years, as for most countries in the world. It should be noted that there is no complete and continuous mapping of Brazilian forest plantations using remote sensing techniques. Therefore, the cited sources provide estimates and not wall-to-wall area determination.

The areas reported by the IBGE from 2014 to 2016 [11] were adopted in this study, except in the case of species other than Eucalyptus and Pinus, as the areas reported by IBÁ were larger than that of IBGE. In other words, we always selected the largest reported area, because IBÁ only reports statistics for its members disregarding independent producers and IBGE is the official governmental institution responsible for forestry statistics in the country. For the period 2010–2013, the areas reported by IBÁ [12] were used, and for the other years, those reported in the FRA2015 [1] were adopted.

### Growth and yield

To estimate carbon stocks, we first estimated the volume yield from productivity data generated through the use of simulators developed by the Brazilian Enterprise on Agricultural Research—Forestry Office (EMBRAPA) [17] (downloaded on February 1, 2018). These computer programs were developed to simulate growth and yield of Eucalypt, Pine, Teak and Acacia´s growth and yield under specific Brazilian climate and soil conditions. They are diameter class bio-mathematical growth models based on the Johnson´s bivariate probability distribution. Tree size distribution (diameter and height) over time are generated by unthinned and thinned stands growing under certain stock (initial density) and site conditions informed by the user. Site and mortality routines drive the simulations. The simulators provide a complete yield table of the stands, including age, stock, mean diameter and height, dominant height, and volumes by grade (specific wood products, such as timber, pulp and paper, firewood and so on). A more detailed description of the rationale of the software are provided by EMBRAPA [17] and OLIVEIRA [18], HAFLEY and BUFORD [19].

1. SISEUCALIPTO: a program developed for Eucalyptus species. In this work, the *Eucalyptus grandis*/*urograndis* version was used, since these are the main species of this genus planted in Brazil. All parameters of the program were kept at default values. The yields per hectare were simulated for the site indices 29, 31, and 33 m (dominant height at age 7 years), representing low, medium, and high productivities, respectively. The simulations were carried out with an initial density of 1111 trees ha^−1^, without thinning, and a 7-year silvicultural rotation was assumed. These parameters correspond to the standard management regime for Eucalyptus in Brazil.
2. SISPINUS: a program developed for Pinus species. In this work, the *Pinus taeda* (Loblolly Pine) version was used since this is the main species planted in Brazil. All input parameters of the program were unchanged, and we simulated the productivities for the site indices 21, 23, and 25 m (dominant height at age 15 years). The simulations were carried out with an initial density of 1111 trees ha^−1^, 50% without thinning and 50% with a thinning at age 8 years (removal of 50% of the initial density, systematic in the sixth row, followed by selective low thinning). A silvicultural rotation of 20 years was also assumed. These are the standards for Pine management in Brazil.
3. SISTECA: a program developed for *Tectona grandis*. All input parameters of the program were kept at default values, and the yields were simulated for site indices 18, 20, and 22 m (dominant height at the age 15 years). The simulations were made with an initial density of 1250 trees ha^−1^, with thinning at age 8 years (removal of 50% of the initial density, only selective), and an assumed silvicultural rotation of 15 years. These are the standards for Teak management in Brazil.
4. SISACACIA: a program developed for Black Wattle (*Acacia mearnsii*). All default input parameters of the program were maintained, and the productivities were simulated for 16, 18, and 20 m (dominant height at age 7 years). The computer simulations were conducted with an initial density of 2000 trees ha^−1^, without thinning, and we assumed a silvicultural rotation of 7 years. These are the standards for Acacia management in the country.

Based on these parameters, we calculated the volume MAI by genus/species as follows in Table 1.

**Table 1:** Commercial volume (MAI) over four cm at the smaller end, estimated by EMBRAPA’s growth and yield simulators for the main tree genera/species planted in Brazil

Genus/species	Commercial volume MAI (m^3^ ha^−1^ years^−1^)		
	Low	Medium	High
*Eucalyptus* ^a^	36	42	48
*Pinus taeda*	22	27	32
*Tectona grandis*	13	17	22
*Acacia mearnsii*	20	25	31

^a^ *E. grandis/urograndis*

Since there is no information on the age structure of forest plantations in Brazil, a uniform rectangular distribution was assumed, i.e., all age classes, from 1 year to the age of rotation, have the same area. Thus, the planted area was divided into *n* parts corresponding to each age, from zero (clear-cut or just-planting) to the rotation age. An additional age class was created to represent any stands older than the age of rotation, with a mean age of 12 years for Eucalypt, 25 years for Pine, 20 years for Teak, and 10 years for Black Wattle. This is an empirically valid assumption for Brazilian plantations.

The volume yields per hectare calculated from the simulations were then multiplied by the areas of each genus/species. In the case of the other species, it was considered that 50% are managed under a multiple-use regime (represented by Teak) and 50% under a pulpwood management regime (represented by Black Wattle). Low productivity was assumed for the period 1990–2000, reflecting the lower technological level during that time. The arithmetic mean of the low and medium productivities was applied for 2000–2010, and the mean of the high and medium productivities was adopted from 2011 onwards, reflecting the breeding techniques that were recently developed.

### Biomass estimation

The total dry biomass (above and below ground) stocked in forest plantations was estimated by the application of the corresponding basic wood density (*BWD*) and biomass expansion factors (*BEF*) to obtain aboveground biomass (AGB), and the root-to-shoot ratio (*R*) for estimating belowground biomass (BGB). *BWD* converts the stem volume into trunk biomass or weight (t), *BEF* expands it to the whole aerial compartment, and *R* considers the root fraction (Eqs. 1, 2, 3, 4, 5, 6):

1 $$B = V*BWD*BEF*(1 + R)$$

where: $B$ = dry total biomass (t ha^−1^); $V$ = commercial stem volume (m^3^ ha^−1^); $BWD$ = basic wood density (t m^−3^);

2 $$BEF = {\text{ biomass expansion factor }} = \frac{AGB}{{B_{stem} }}\left( {\text{dimensionless}} \right)$$

$AGB$ = dry aboveground biomass (t ha^−1^);

3 $$B_{stem} = {\text{ dry stem biomass }} = V*BWD\,\left( {\text{t}} \right)$$

4 $$R = {\text{ root-to-shoot ratio }} = \frac{BGB}{AGB}\left( {\text{dimensionless}} \right)$$

$BGB$ = dry belowground biomass (t ha^−1^); and

5 $$AGB = V*BWD*BEF$$

6 $$BGB = B - AGB$$

The values used in this work were obtained from the literature (Table 2). In some cases, single values were applied for each genus/species, whereas in others an age differentiation was applied (when available), with the variations shown in the body of the table.

**Table 2:** Parameters for biomass and carbon estimation for tree species planted in Brazil and the source references

Genus/species	*BWD* (t m^−3^)	*BEF* (t t^−1^)	*R* (t t^−1^)	*BCF* (g g^−1^)
*Eucalyptus*	0.5090	1.0365–1.2970	0.1700	0.4630
*Pinus*	0.3223–0.3782	1.0902–2.5463	0.1097–0.5830	0.4536
*T. grandis*	0.4750	1.4100	0.4800	0.5000
*A. mearnsii*	0.6345	1.2468	0.1309	0.4410

Range refer to age-dependent values

*BWD* basic wood density, sources: [20–23]

*BEF* biomass expansion factor, sources: [24–27]

*R* root-to-shoot ratio, sources: [20, 21, 25, 27]

*BCF* carbon fraction, sources: [20, 26–29]

To calculate the total biomass, both *AGB* and *BGB* for all forested land, the results of Eqs. 1, 5, and 6 (per hectare basis) were multiplied by the plantation areas of each genus/species in each year of the analysis.

### Carbon stock estimation

To estimate the carbon stocked in the tree tissues, the *AGB* and *BGB* of each genus/species were multiplied by the respective carbon fractions (*CF*) (Eqs. 7, 8 and 9). A similar procedure was applied to calculate the carbon stock in necromass (Eq. 10), with unit area values taken from various studies carried out for the target species, by genus/species and age (Table 3). For biomass estimation, the carbon fractions are those shown in Table 2, and for necromass, *CF* values are given in Table 3.

**Table 3:** Deadwood (*DW*), litter (*L*) and carbon fraction (*NCF*) values used to estimate carbon stock in necromass of forest plantations in Brazil, and source references

Age (years)	Necromass (t ha^−1^)							
	*Eucalyptus*		*Pinus*		*T. grandis*		*A. mearnsii*	
	Deadwood	Litter	Deadwood	Litter	Deadwood	Litter	Deadwood	Litter
1	0.29	1.10	1.84	6.38	0.00	0.00	0.07	1.91
2	0.62	2.37	2.03	7.04	0.05	1.11	0.15	3.82
3	0.95	3.63	2.22	7.70	0.12	2.84	0.22	5.73
4	1.28	4.90	2.41	8.36	0.19	4.57	0.29	7.65
5	1.61	6.16	2.60	9.02	0.27	6.30	0.40	10.31
6	1.94	7.43	2.79	9.69	0.34	8.03	0.50	12.97
7	4.11	6.85	2.98	10.35	0.41	9.76	0.73	18.87
> 7	5.73	10.01	–	–	–	–	0.78	20.35
8	–	–	3.17	11.01	0.49	11.48	–	–
9	–	–	3.36	11.67	0.56	13.21	–	–
10	–	–	3.55	12.33	0.63	14.94	–	–
11	–	–	3.74	12.99	0.71	16.67	–	–
12	–	–	3.93	13.65	0.78	18.40	–	–
13	–	–	4.12	14.31	0.85	20.13	–	–
14	–	–	4.31	14.97	0.93	21.86	–	–
15	–	–	4.50	15.63	1.00	23.59	–	–
>15	–	–	–	–	–	–	–	–
16	–	–	4.69	16.29	1.36	32.23	–	–
17	–	–	4.88	16.95	–	–	–	–
18	–	–	5.07	17.61	–	–	–	–
19	–	–	5.26	18.27	–	–	–	–
20	–	–	5.45	18.93	–	–	–	–
>20	–	–	6.40	22.24	–	–	–	–
*NCF* (g g^−1^)								
	0.4251	0.4251	0.4030	0.4030	0.4330	0.4330	0.4330	0.4330

*Eucalyptus* source: [30–32]

*Pinus* source: [33–35]

*T. grandis* sources: [36–38]

*A. mearnsii* sources: [39, 40]

7 $$CAGB = AGB*BCF$$

8 $$CBGB = BGB*BCF$$

9 $$CB = CAGB + CBGB$$

where: $CAGB$ = carbon stock in aboveground biomass (t ha^−1^);$CBGB$ = carbon stock in belowground biomass (t ha^−1^); $CB$ = carbon stock in total biomass (t ha^−1^);

10 $$CDW = DW*NCF$$

11 $$CL = L*NCF$$

12 $$CN = CDW + CL$$

where: $CDW$ = carbon stock in deadwood (t ha^−1^); $CL$ = carbon stock in litter (t ha^−1^); $CN$ = carbon stock in necromass (t ha^−1^); $DW$ = deadwood (t ha^−1^); $L$ = litter (t ha^−1^).

Hence, the unit area values for carbon stocks were multiplied by the respective area in hectares of each genus/species to generate the values for total forested land.

To calculate the carbon stock of HWP, first we took statistics for sawnwood, wood-based panels, and treated wood (stacks, poles, and sleepers) produced from tree plantations in cubic meters, and for the historic consumption of logs [13, 14, 41–43], called here solid wood. Other HWP (such as pulp and paper, firewood) were not considered because they do not store C in the long term. Then, we converted roundwood into product by applying the apparent wood density of each product, and species-specific values, to obtain the mass of each product in tonnes. For each type of HWP, the mass in tonnes was multiplied by the volume and the carbon fraction (Table 4), to calculate the carbon stocked in solid wood products. Since no specific information of the Brazilian HWP are available, the lifetime of each product was taken from IPCC reports (default values). Further confirmation of these values is needed.

**Table 4:** Conversion of roundwood to HWP, apparent wood density and carbon fraction of HWP from tree species planted in Brazil, and source references

Product	*AWD* (t m^−3^)	*HWPCF* (g g^−1^)
Sawnwood	0.458^a^	0.5000^a^
Plywood	0.542^b^	0.4930^b^
Particleboard	0.596^c^	0.4540^c^
Pig iron	NA	0.0400

^a^[48]

^b^[49]

^c^[50]

Secondly, to calculate carbon stocked in pig iron, we multiplied the official statistics for the mass of charcoal coming exclusively from forest plantations [13, 44] and consumed in pig iron production [45, 46], and the carbon content in the product (Table 4). Pig iron is the product of smelting iron ore in blast furnaces with a high-carbon fuel and reductant such as charcoal as a fuel and reductant. We did not consider pig iron produced by using coal, coke or charcoal made from deforestation in this analysis.

Exports of HWP were discounted from the calculations [12, 47] and imports were neglected because Brazil is not a significant importer of wood products.

13 $$CHWP = CSW + CPI$$

14 $$CSW = SW*HWPCF$$

15 $$CPI = PI*HWPCF$$

where: $CHWP$ = carbon stock in HWP (t); $CSW$ = carbon stock in solidwood products (t); $CPI$ = carbon stock in pig iron (t); $SW$ = solidwood produced excluding exports and imports (t); $HWPCF$ = HWP carbon fraction (g g^−1^); $PI$ = pig iron produced excluding exports and imports (t).

### CO_2_ removals

Gross carbon dioxide atmospheric removals were calculated by stoichiometry, assuming the atomic masses: C = 12 and O = 16, therefore CO_2_ = 44, as follows (Eq. 5):

16 $$CO_{2} eq = C*\frac{44}{12}$$

### CO_2_ removals

Gross carbon dioxide atmospheric removals were calculated through stoichiometry, assuming the atomic masses C = 12 and O = 16. Therefore CO_2_ = 44, as follows (Eq. 5):

## Results

### Extent of forest plantations

The area of planted forests in Brazil increased from 4934 to 10,212 million hectares (Mha) from 1990 to 2016, which means that it has more than doubled during the 26-year period of study (Table 4). Since the beginning of reforestation in Brazil, Eucalyptus has been the dominant planted tree in Brazil, and its proportion increased from 60% in 1990 to 74% in 2016. Eucalyptus experienced significant growth in its planted area compared to Pinus, which increased by only 17%. Pinus has been losing area to other more competitive tree species in Brazilian forestry and decreased from 36% of the planted area in 1990 to 20% in 2016, a remarkable change. While the other species, such as *Tectona grandis* and *Acacia* spp. have a small proportion of the total planted forest area, they have increased their relative proportion from 4% in 1990 to 6% in 2016.

### Volume stock

The commercial volume of stems (standing volume of the logs from a 4-cm threshold diameter with bark) increased from 774 Mm^3^ in 1990–1999 Mm^3^ in 2016, an increase of 158% (Table 5). Eucalyptus accounted for 52% of the volume stock in 1990, increasing to 67% in 2016. Pinus decreased sharply as a proportion of volume stock, from 46% in 1990 to 28% in 2016, and the other species increased from 3 to 4%. Two factors affected the growth of the volume stock, namely the increase in planted area and higher productivity achieved by plantations in recent years due to improvements in genetics and silviculture practices. Hence, the increase in volume was proportionally greater than the forest extent in hectares.

**Table 5:** Main quantitative estimates for Brazilian forest plantations by year

Variable	Genus/species	Year									
		1990	2000	2005	2010	2011	2012	2013	2014	2015	2016
Area (ha)	*Eucalyptus*	2,964,000	2,965,880	3,642,719	4,900,949	5,049,714	5,304,164	5,473,176	6,952,509	7,444,625	7,543,707
	*Pinus*	1,769,000	1,840,050	1,831,485	1,756,359	1,641,892	1,562,782	1,570,146	2,049,234	2,065,560	2,079,162
	Others	201,141	319,976	326,176	462,390	489,282	521,131	557,652	588,521	589,201	589,361
	All	4,934,141	5,125,906	5,800,380	7,121,708	7,182,899	7,390,089	7,602,987	9,590,264	10,099,386	10,212,230
Commercial stem volume (m^3^)	*Eucalyptus*	400,370,533	400,624,480	547,723,276	736,912,137	899,438,225	944,760,011	974,863,865	1,238,357,728	1,326,011,790	1,343,659,945
	*Pinus*	353,192,911	367,378,528	409,089,231	392,308,729	447,805,519	426,229,256	428,237,695	558,902,958	563,355,671	567,065,446
	Others	20,588,655	32,752,524	38,413,700	54,455,602	73,464,373	78,246,415	83,729,944	88,364,841	88,466,942	88,490,965
	All	774,152,099	800,755,532	995,226,207	1,183,676,468	1,420,708,118	1,449,235,681	1,486,831,504	1,885,625,528	1,977,834,402	1,999,216,356
Above ground biomass (t)	*Eucalyptus*	224,193,381	224,335,582	306,422,510	412,263,777	503,107,907	528,459,007	545,297,837	692,685,220	741,715,214	751,586,850
	*Pinus*	154,766,294	160,982,318	179,425,835	172,065,936	196,722,348	187,243,828	188,126,142	245,527,795	247,483,886	249,113,602
	*Outros*	14,947,239	23,778,134	27,877,360	39,519,194	53,277,114	56,745,100	60,721,812	64,083,086	64,157,130	64,174,553
	All	393,906,914	409,096,033	513,725,705	623,848,906	753,107,369	772,447,936	794,145,790	1,002,296,102	1,053,356,231	1,064,875,004
Below ground biomass (t)	*Eucalyptus*	38,112,875	38,137,049	52,091,827	70,084,842	85,528,344	89,838,031	92,700,632	117,756,487	126,091,586	127,769,765
	*Pinus*	20,704,449	21,536,021	24,077,728	23,090,080	26,539,192	25,260,475	25,379,505	33,123,381	33,387,271	33,607,131
	*Outros*	4,539,300	7,221,139	8,488,602	12,033,518	16,300,512	17,361,567	18,578,270	19,606,676	19,629,330	19,634,661
	All	63,356,624	66,894,209	84,658,157	105,208,440	128,368,048	132,460,073	136,658,408	170,486,545	179,108,188	181,011,557
Deadwood mass (t)	*Eucalyptus*	5,438,011	5,441,460	6,683,247	8,991,705	9,264,642	9,731,478	10,041,562	12,755,674	13,658,553	13,840,337
	*Pinus*	6,373,981	6,629,985	6,599,124	6,328,433	5,915,991	5,630,945	5,657,479	7,383,707	7,442,532	7,491,542
	*Outros*	116,416	185,195	188,784	267,621	283,186	301,619	322,757	340,623	341,017	341,109
	All	11,928,407	12,256,640	13,471,155	15,587,759	15,463,819	15,664,043	16,021,798	20,480,005	21,442,102	21,672,989
Litter mass (t)	*Eucalyptus*	13,978,429	13,987,295	17,179,314	23,113,214	23,814,800	25,014,804	25,811,876	32,788,512	35,109,365	35,576,643
	*Pinus*	22,144,728	23,034,147	22,926,929	21,986,485	20,553,562	19,563,246	19,655,430	25,652,758	25,857,131	26,027,403
	*Outros*	2,161,921	3,439,193	3,505,832	4,969,899	5,258,942	5,601,264	5,993,802	6,325,591	6,332,900	6,334,619
	All	38,285,077	40,460,635	43,612,075	50,069,598	49,627,304	50,179,313	51,461,108	64,766,861	67,299,396	67,938,666
Solidwood* (t)	All	8,552,715	10,164,398	11,886,870	12,673,541	12,047,222	11,922,273	12,173,789	11,601,964	9,749,963	8,916,910
Pig iron (t)	All	3,017,544	3,206,140	4,300,000	800,000	2,200,000	1,800,000	4,600,000	6,700,000	7,100,000	7,900,000

### Biomass stocks

Biomass stocked in Brazilian forest plantations increased by 172% during the study period, from 457 Mt in 1990 to 1246 Mt in 2016 (Table 5). This refers to the total dry biomass (AGB and BGB) stored in the stems, branches, leaves, and roots of the trees, excluding necromass (dead tissues), and soil organic carbon. AGB accounted for 86% of the total biomass, and BGB for 14%. Eucalyptus accounted for 57% of the biomass stock in 1990 and 71% in 2016, whereas Pinus fell from 38 to 23% in this period. The other species increased from 4 to 7%.

### Necromass stocks

Necromass on the ground increased from 50 Mt in 1990 to 90 Mt in 2016, of which deadwood accounted for 24% and litter for 76%. Eucalyptus accumulated 55% of the wood debris in the forest plantations, whereas Pinus and the other species accounted for 37 and 7%, respectively (Table 5).

### Harvested wood products

Solid HWP (sawnwood, wood-based panels, stacks, poles, and sleepers) production, excluding exports and imports, increased from 1990 to 2013 and then decreased. Pig iron production increased continuously, growing 264% in 26 years (Table 5).

### Carbon stocks

**Fig. 1:**
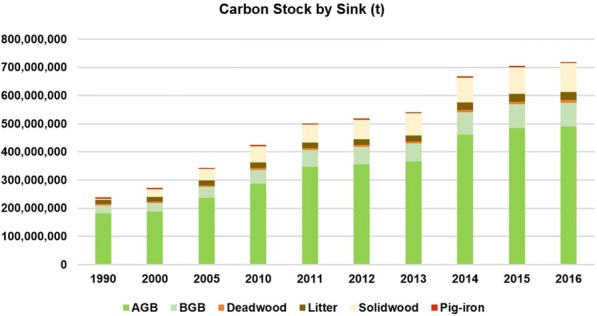
Dynamics of carbon stocks in the Brazilian forest plantations by sink during 1990–2016

In 1990 the Brazilian forest plantations stocked 231 Mt C (biomass and necromass), and the HWP produced stored 2.2 Mt C. In 2016 these values increased to 612 Mt C and 76 Mt C, considering the cumulative wood from forest plantations produced over the 26-year period. This corresponds to an increase of 295% in C stocking. This large increase was due to a strong expansion in forest area and higher productivity of the stands, as well as the accumulation of C in products that remained in use inside the country during the accounting period. In this calculation, we did not consider the C storage in products produced before 1990 due to a lack of reliable data (Fig. 1).

In 2016, AGB represented 71% of the carbon stock in forest and products, whereas BGB accounted for 12%. Deadwood and litter stocks were 1.32 and 4.09%, respectively. Woody products accounted for 11% of the total carbon quantified, 10.44% was stocked in solid wood materials and less than 0.59% in pig iron. This is because sawnwood, wood-based panels, stacks, and poles are mostly composed of wood with a carbon fraction of 40–50%, while the carbon content in pig iron is low, only 4%.

**Fig. 2:**
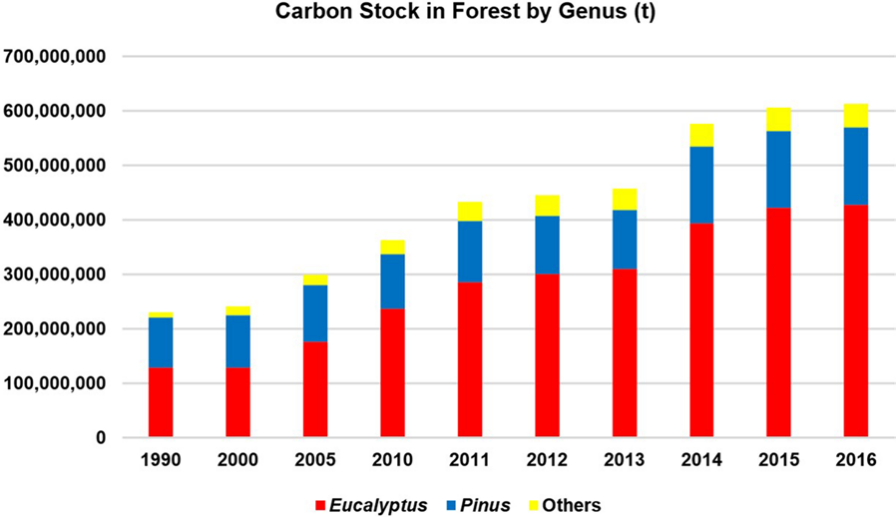
Dynamics of carbon stocks in the Brazilian forest plantations by genus during 1990–2016

Eucalypt plantations have great importance in Brazilian forestry and also demonstrate a remarkable potential for carbon storage. They accounted for 56% of the total carbon stock in forest in 1990 and 70% in 2016. The other species have also continuously increased in carbon storage, from 4% in 1990 to 7% in 2016, due to diversification of plantations designed to produce more valuable woody products. Conversely, carbon stocks in Pinus stands increased much slower, and Pinus drastically decreased as a share of total carbon stock in forest from 38% in 1990 to 22% in 2016 (Fig. 2).

The carbon storage in forest products could not be calculated accurately by genus because there is no data available for this purpose, particularly for exports. The exact proportion of sawnwood, wood-based panels, stacks, poles, and sleeper production by species is not clear because there many small and independent producers throughout the country and also because institutions compute the data in aggregate. However, it is reasonable to assume that almost all wood from plantations used for pig iron production comes from Eucalyptus stands.

### CO_2_ removals

The growth of total carbon stocks in Brazilian forest plantations and wood products corresponds to the gross removal (disregarding emissions) of 1.7 Gt CO_2-_eq. (carbon dioxide equivalent), applying the respective atomic masses. Carbon in biomass accounted for 80% (1338 Gt) of this removal, necromass 3.64% (60.7 Mt) and HWP for 16% (270.1 Mt). The expansion of the planted area, the increased productivity of the stands, and the woody product sink were responsible for this GHG mitigation effect.

## Discussion

### Extent of forest plantations

The 1960s marked the beginning of the Brazilian Federal Government’s fiscal incentives policy for reforestation, which expanded in the 1970s and was interrupted in 1988 [51]. There was an abrupt jump in the area planted, from 500,000 ha in the late 1960s to 2.6 Mha in 1975, and over 6 Mha in 1985 [52]. The end of the subsidies caused an immediate reduction in annual planting rates, and the area of forest decreased to 5 Mha in the early 1990s. However, there was a resumption of plantation growth using private resources from 1995, and for nearly a quarter of a century the country has experienced a continuous increase in plantation area.

Since the beginning of plantation silviculture in Brazil until the present day, no primary survey by either the public or private sectors covering the entire area of planted forests in Brazil has been carried out. Even the National Forest Inventory, completed in the 1970s, was unable to give a complete overview of the extent of these forests. The new National Forest Inventory, restarted in the early 2000s, has the enormous challenge of providing reliable and updated forest statistics for the country. However, the focus appears to be on natural forests, which are much larger than plantations in area, but less relevant to the forest-based economy. The consequence of the lack of actual forest cover data from remote sensing and field measurements is the persistent use of approximations and rough estimates, with no perspective for change in the short term. This is a negative for the country’s socioeconomic development, both for public policy and private forestry investments.

The expansion of the area of planted forests since 1990 can be considered very successful and coincides with the recent construction and operation of large pulp and paper mills in Mato Grosso do Sul, Bahia, and Maranhao States, providing economic growth opportunities, jobs, and income in the forestry sector, which was previously less established in these regions of the country. On the other hand, the poorly planned expansion of productive forests, especially in pioneer regions, can bring concerns and potential losses. Expansions of the forest base should be preceded by competent planning, thorough knowledge of the production chain (internal and external), use of scientific information, and application of appropriate technologies for forest management, as well as consideration of socio-environmental externalities, risks, and uncertainties in a country with frequent instabilities.

### Volume of biomass and necromass

The expansion of forest plantations since 1990 has led to the formation of a large wood stock in Brazil, over 2.1 Gm^3^, currently accounting for more than 80% of consumption by national forestry companies. This volume, calculated for 2015, is close to that reported in FRA2015 [1], despite the different methodological approaches used in each study. It is noteworthy that the FAO report makes no reference to whether the volume is only commercial, or the total used, and the threshold diameter considered for the calculations is not provided. In this study, we used four cm as the threshold limit.

On the other hand, a significant difference of more than 50% was noticed between the calculated biomass, which was 1.246 Mt in this study and reported as 1.896 Mt in the FRA2015. The main cause was identified as the difference in *BEF* and *R* values used in each study. Although the expansion factors for plantations are not explicit in FRA, they could be deducted from the numbers in the report. FRA used 1.50 as an average *BEF* for all species, while we utilized species-specific values (plus age-specific values in some cases), which resulted in an overall weighted mean *BEF* of 1.27. In addition, FRA used 0.20 as an average *R* value for all plantations, whereas in this study we utilized species-specific root-to-shoot ratios, which resulted in an overall mean of 0.17. Since most of the available literature reporting expansion factors support the values used in this study, we believe that FRA’s biomass estimates are overestimated and should be revised in future reports. Necromass (deadwood and litter) is not reported for plantations by the Brazilian FRA2015 and comparisons are not possible.

### Carbon stocks

The difference between this study and FRA regarding biomass has direct implications for carbon stocks. However, this is not the only factor, since the carbon fractions used in each case are also different. FRA used 0.47 (the IPCC default) for all species, and we used species-specific values, ranging from 0.44 to 0.50, with a weighted mean of 0.46. The combination of these factors resulted in a difference of 57% between the studies, with 569 Mt calculated in this study (in 2015) and 891 Mt reported by FRA. Again, the literature supports our estimates, and hence we believe that FRA’s carbon estimates are overestimated and should be revised.

Although plantation necromass estimates are not provided by FRA, litter carbon figures are provided. FRA used an overall value of 22 t ha^−1^ for all species and ages. On the contrary, we adopted species and age-specific values (Table 4) supported by the literature. Furthermore, species-specific carbon values for necromass were applied in this study. FRA’s litter carbon values are much higher than those estimated in this study. While the FRA reported 171 Mt, our estimates showed only 37 Mt. As the literature indicates much lower values for litter stocks in the Brazilian forest plantations, we can state that the estimates in the FRA report are strongly inflated and need careful revision. This divergence in estimates demonstrates the need for more research on expansion factors and other parameters used for biomass and carbon quantification, and systematization of methods is required for large-scale studies.

Forest products also play an important role as a carbon sink. In this study, HWP produced and transformed into materials represents almost 11% of the total carbon stocked in 2016 in forest plantations in Brazil. Sawnwood, wood-based panels, stacks, poles and sleepers, and pig iron also retain carbon inside their structure for a long time and contribute to avoided emissions from other carbon-intensive products. In this study, we did not consider the GHG emissions due to timber harvesting or processing of these products. These demand further consideration and should be included in future studies.

### CO_2_ removals

The dynamics of total carbon stocked in Brazilian forest plantations and HWP over the 26 years corresponds to the removal of 1.7 Gt CO_2-_eq. Removals by forest stands, due to the expansion of the planted area and increases in stand productivities, accounted for 84% (1.4 Gt) of this removal, and the wood product sink for 16% (270 Mt). We did not consider removals by the stands (trees and organic matter) and HWP before 1990. The figures mentioned are gross removals because the corresponding anthropic emissions were not considered. FRA2015 does not consider carbon stocks in wood products, and therefore no comparisons can be made.

Since 1990, Brazil emitted more than 50 Gt CO_2-_eq. from the AFOLU sector. This sector was responsible for more than 80% of the accumulated emissions during that period. Most of these emissions were due to deforestation, especially in the Amazon Forest biome. However, forest plantations traditionally have not been established in the Amazonia region. Most plantations are located in regions that suffered anthropization a long time ago, such as the Mata Atlantica biome, where the current environmental legislation does not allow conversion from native forest to industrial plantations. Therefore, the impact of forest plantations on deforestation and GHG emissions in this period is small compared to agriculture and livestock. In contrast, planted forests have contributed to climate change mitigation through carbon sequestration, as demonstrated in this study. In addition, Brazilian foresters have an extensive area of native forest, protected by legislation that determines a percentage of natural vegetation to be maintained on each property, depending on the biome. This also helps alleviate, in part, criticisms of forest monocultures.

The GHG removals by Brazilian planted forests and their products during the 26-year study period correspond approximately to the sum of the country’s emissions over that period from the waste sector and exceed the national AFOLU emissions for 2016. Moreover, this sequestration is equivalent to approximately 3% of Brazil’s total GHG emissions over the 26 years, according to data published by the Brazilian Climate Observatory [16]. This is the only forest typology that increased in area in the country [15], becoming a relevant C sink for the Brazilian emission balance [15].

The CO_2_ mitigation calculated here should also be increased by calculating avoided CO_2_ emissions through bioenergy use with wood replacing fossil fuels, which was not considered in this study. In addition, the increase in organic matter by forest plantations can also mean higher C stocked in the soil. Complementary studies should consider these aspects, as well as the emissions from this sector, to improve the quality of Brazil’s GHG inventories and contribute to meeting Brazil’s commitments to its NDC and the Paris Agreement.

## Conclusions

Carbon stocks in Brazilian forest plantations increased from 231 Mt in 1990 to 612 Mt in 2016. In addition, plantation wood products stocked 74 Mt C in their structures. This sequestration was due to the increased forest area and higher productivity of the stands, as well as the accumulation of carbon by HWP over the 26 years. Eucalyptus plantations play the primary role in Brazilian forestry and in carbon storage, followed by Pinus plantations. Carbon dioxide removals by the Brazilian forest plantations over the study period correspond to the totality of the country’s emissions from the waste sector within the same period, or the AFOLU emissions in 2016. Forest plantations play an important role in mitigating GHG emissions in Brazil, and the estimates provided by this study can improve the accuracy of the Brazilian GHG inventory and help to accomplish the goals of Brazil’s NDC and the Paris Agreement.

## Abbreviations

AFOLU: agriculture, forestry, and other land use
AGB: aboveground biomass
BEF: biomass expansion factor
BGB: belowground biomass
BWD: basic wood density
C: carbon
CF: carbon fraction
CO_2-_eq.: carbon dioxide equivalent
DW: deadwood
EMBRAPA: Brazilian Enterprise on Agricultural Research—Forestry Office
FAO: Food and Agriculture Organization
FRA: Forestry Resource Assessment
GHG: greenhouse gases
HWP: harvested wood products
MAI: mean annual increment
IBA: Association of Brazilian Forestry Industries
IBGE: Brazilian Institute on Geography and Statistics
N: necromass
NCF: necromass carbon fraction
NDC: Nationally Determined Contribution
SFB: Brazilian Forest Service
